# Decreased, Deformed, Defective—How HIV-1 Vpu Targets Peroxisomes

**DOI:** 10.1128/mBio.00967-20

**Published:** 2020-05-26

**Authors:** Kristina Hopfensperger, Daniel Sauter

**Affiliations:** a Institute of Molecular Virology, Ulm University Medical Center, Ulm, Germany

**Keywords:** Vpu, human immunodeficiency virus, miRNA, peroxisomes

## Abstract

Peroxisomes are found in essentially all eukaryotic cells and have been described as important hubs in innate sensing and the induction of type III interferons upon viral infection. Nevertheless, it remains poorly investigated how viral pathogens modulate biogenesis or function of peroxisomes to evade innate sensing and restriction. In a recent study, Hobman and colleagues found that the accessory viral protein u (Vpu) of HIV-1 inhibits peroxisome activity by depleting cellular peroxisome pools. This depletion could be ascribed to a Vpu-dependent induction of four microRNAs (miRNAs) that suppress the expression of peroxisomal biogenesis factors PEX2, PEX7, PEX11B, and PEX13. Although the downstream effects on antiretroviral gene expression and HIV-1 replication remain to be determined, these findings provide important insights into peroxisome biogenesis and the modulation of cell organelles by HIV-1 Vpu.

## Abstract (precis)

Peroxisomes are found in essentially all eukaryotic cells and have been described as important hubs in innate sensing and the induction of type III interferons upon viral infection. Nevertheless, it remains poorly investigated how viral pathogens modulate biogenesis or function of peroxisomes to evade innate sensing and restriction. In a recent study, Hobman and colleagues found that the accessory viral protein u (Vpu) of HIV-1 inhibits peroxisome activity by depleting cellular peroxisome pools.

## COMMENTARY

**Peroxisomes play opposing roles in viral infection.** Peroxisomes are small organelles that exert a plethora of functions, including different pathways in lipid metabolism and the reduction of reactive oxygen species (ROS). Initially described as *microbodies*, the term *peroxisome* was coined in the late 1960s, upon the discovery that these organelles harbor several oxidases involved in the production of hydrogen peroxide (H_2_O_2_). More than 40 years later, it became clear that peroxisomes also serve as important platforms for innate antiviral signaling cascades. Upon sensing of viral nucleic acids by RIG-I-like receptors, the adaptor protein mitochondrial antiviral-signaling protein (MAVS) forms a signaling complex that mediates the expression of interferons (IFNs) and other antiviral factors. Intriguingly, MAVS are not only located in the outer membrane of mitochondria, but also in peroxisomal membranes. In response to RNA virus infection, these peroxisomal MAVS are essential for a rapid induction of antiviral gene expression, independently of type I IFNs (1). A follow-up study identified peroxisomes as a key organelle in the induction of type III IFN expression (2). The selection pressure exerted by this antiviral pathway is reflected by the evolution of evasion strategies in several viruses. For example, West Nile and Dengue viruses reduce peroxisome numbers and therefore type III signaling by inducing the degradation of peroxisomal biogenesis factor 19 (PEX19) (3). Similarly, Zika virus (ZIKV) nonstructural protein 2A (NS2A) interacts with PEX19 and PEX3, and peroxisome density is decreased in ZIKV-infected cells (4). Finally, hepatitis C virus uses its protease to degrade MAVS on both peroxisomes and mitochondria (5). As recently reviewed by Cristea and colleagues (6), some viruses may also exploit the metabolic pathways of peroxisomes for their own purposes. For example, viruses such as human cytomegalovirus, West Nile virus, and influenza virus harbor specific ether lipids (i.e., plasmalogens) in their virion envelopes that are exclusively synthesized in peroxisomes. Consistent with this, several herpesviruses alter peroxisome composition and fail to infect cells from Zellweger syndrome patients that entirely lack peroxisomes (6). Thus, peroxisomes may exert both pro- and antiviral activities that can be targeted by viruses to enhance viral replication.

**FIG 1:**
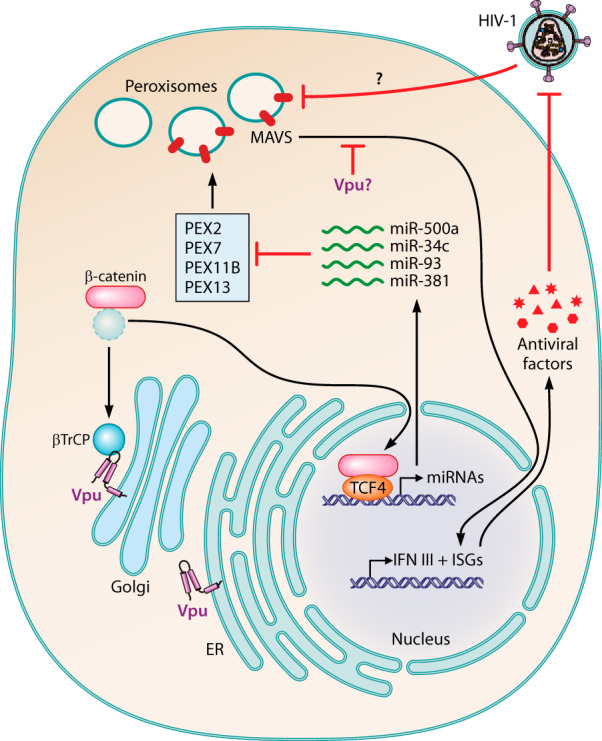
HIV-1 Vpu suppresses peroxisome biogenesis. The HIV-1 accessory protein Vpu induces several miRNAs (miR500a, miR-34c, miR-93, and miR-381) that suppress the expression of peroxisomal biogenesis factors PEX2, PEX7, PEX11B, and PEX13 (8). Vpu stabilizes β-catenin by sequestering the adapter protein βTrCP, which usually recruits E3 ubiquitin ligase complexes and induces the degradation of β-catenin (10). As a result, Vpu may enhance β-catenin-dependent activation of the transcription factor TCF-4, which drives expression of miR500a, miR-34c, miR-93, and miR-381 (8). Whether Vpu-mediated suppression of peroxisome biogenesis inhibits peroxisomal MAVS signaling and expression of type III interferon (IFN III) or interferon-stimulated genes (ISGs) remains to be determined.

**HIV-1 Vpu depletes cellular peroxisomes.** In 2017, Hobman and colleagues (7) added the human immunodeficiency virus type 1 (HIV-1) to the list of pathogens interfering with peroxisome biogenesis factors (Fig. 1). They found that the expression of PEX2, PEX7, PEX11B, and PEX13 is significantly reduced in brain tissues of HIV-1 infected individuals and *ex vivo* infected macrophages (7). These factors play key roles in the biogenesis, division, and/or translocation machinery of peroxisomes. In their current follow-up study (8), Hobman and colleagues deciphered the underlying mechanism, demonstrating that the HIV-1 accessory protein Vpu is necessary and sufficient to suppress the expression of PEX2, PEX7, PEX11B, and PEX13. As a result, Vpu depletes cellular peroxisome pools by about 30%. It is tempting to speculate that the Vpu-induced decrease in peroxisome numbers may contribute to HIV-associated neurocognitive disorders, since loss of peroxisomes is associated with neurodegeneration and premature aging (7). It should be noted that the authors solely relied upon the Vpu protein of the lab-adapted HIV-1 strain NL4-3 in their experiments. Since this Vpu is known to have lost several immune-evasion activities upon passaging in cell lines, it will be interesting to investigate whether the ability to suppress PEX expression is conserved or potentially even more pronounced in primary HIV-1 strains, including brain-derived isolates.

**Vpu inhibits peroxisome biogenesis in a β-catenin- and miRNA-dependent manner.** Vpu is a multifunctional protein that exerts several immune-evasion activities in HIV-1-infected cells (reviewed in reference 9). For example, Vpu enables the release of fully infectious viral particles by counteracting the host restriction factor tetherin and down-modulating the primary HIV-1 receptor CD4. Furthermore, Vpu suppresses antiviral gene expression by inhibiting the activation of the host transcription factor NF-κB. All these Vpu functions depend on its ability to bind the adapter protein β-transducin repeat-containing protein (βTrCP) and subsequent recruitment of cellular ubiquitin ligase complexes. For example, sequestration of βTrCP has been shown to result in a stabilization of βTrCP-binding proteins such as IκB or β-catenin (10). While stabilization of the NF-κB inhibitor IκB suppresses antiviral gene expression, the Hobman lab uncovered how Vpu-mediated β-catenin stabilization may modulate peroxisome biogenesis. Using small interfering RNA (siRNA)-mediated knockdown, they show that β-catenin and its downstream effector T-cell factor 4 (TCF-4) are required for the suppression of PEX2, PEX7, PEX11B, and PEX13 expression by HIV-1 Vpu in HeLa cells. Intriguingly, they found predicted TCF-binding sites upstream of four miRNA genes (*miR500a*, *miR-34c*, *miR-93*, and *miR-381*) regulating the expression of peroxisome biogenesis factors PEX2, PEX7, PEX11B, and PEX13, respectively (8). Consistent with this, Vpu induced the expression of these four miRNAs, but had no effect on a control miRNA (miR-483) lacking putative TCF-binding sites. Notably, these PEX-targeting miRNAs (miR-500a, miR-34c, miR-93, and miR-381) were previously also found to be upregulated in brain tissue from HIV-1 infected individuals and primary macrophages (7). Although direct evidence for a role of these miRNAs in Vpu-mediated peroxisome modulation is missing, the proposed cascade of Vpu>β-catenin>TCF4>miRNA>PEX is compelling and convincing. In agreement with a β-catenin-dependent pathway, the authors also found that the adaptor protein βTrCP and the respective binding sites in Vpu are required for efficient suppression of PEX expression and peroxisome depletion. Thus, Hobman and colleagues not only identified a novel function of Vpu with a potential role in HIV-1 pathogenicity, but may have also uncovered a novel β-catenin- and miRNA-dependent pathway regulating peroxisome biogenesis.

**Future perspectives.** While the effects of HIV-1 Vpu on PEX protein expression and peroxisome numbers are conclusive, the downstream effects on peroxisome activity remain less clear. In a first approach, the Hobman lab already monitored the impact of Vpu on cellular levels of nonesterified fatty acids (NEFAs), which are, among others, metabolized by peroxisomes. Consistent with the observed depletion of peroxisome numbers, they found that Vpu induced an accumulation of NEFAs (8). Importantly, this effect was not observed upon knockout of PEX19 and is therefore likely to be peroxisome-dependent. In future experiments, it will be important to test whether Vpu also prevents peroxisome-dependent MAVS signaling and the downstream induction of type III interferon expression. One challenge will be to ascribe observed effects to specific Vpu functions, since its ability to suppress peroxisome biogenesis, to inhibit NF-κB, and to antagonize tetherin-mediated sensing may all reduce antiviral gene expression. Selective mutants will help to define the relative contribution of individual Vpu functions to immune evasion. Together with knockdown or knockout studies, these mutants will also help to address whether the pro- or antiviral activities of peroxisomes prevail.

Notably, the findings by Hobman and colleagues also have implications beyond HIV/AIDS. The observation that βTrCP depletion results in lower peroxisome numbers suggests that other viral proteins which sequester βTrCP may share the anti-peroxisome activity of HIV-1 Vpu. One promising candidate is the poxvirus protein A49, which activates Wnt/β-catenin signaling by targeting βTrCP (11). Finally, the study by the Hobman lab also provides a fruitful basis for further projects that aim at characterizing intra- and extracellular stimuli that modulate peroxisome function by inducing β-catenin signaling and the expression of PEX-targeting miRNAs. Their study provides a nice example of how elucidating virus-host interactions improves our understanding of basic cellular pathways and may ultimately suggest new therapeutic approaches for other peroxisomal diseases such as Zellweger spectrum disorders.
